# Fabrication of Novel Pre-Polymerized BisGMA/Silica Nanocomposites: Physio-Mechanical Considerations

**DOI:** 10.3390/jfb14060323

**Published:** 2023-06-17

**Authors:** Ali Alrahlah, Rawaiz Khan, Abdel-Basit Al-Odayni, Waseem Sharaf Saeed, Leonel S. Bautista, Sajjad Haider, Merry Angelyn Tan De Vera, Abdulrahman Alshabib

**Affiliations:** 1 Restorative Dental Sciences Department, College of Dentistry, King Saud University, Riyadh 11545, Saudi Arabia; abdalshabib@ksu.edu.sa; 2 Engineer Abdullah Bugshan Research Chair for Dental and Oral Rehabilitation, College of Dentistry, King Saud University, Riyadh 11545, Saudi Arabia; aalodayni@ksu.edu.sa (A.-B.A.-O.); wsaeed@ksu.edu.sa (W.S.S.); lbautista@ksu.edu.sa (L.S.B.); 3 Department of Chemical Engineering, College of Engineering, King Saud University, Riyadh 11421, Saudi Arabia; shaider@ksu.edu.sa; 4 Research Center, College of Dentistry, King Saud University, Riyadh 11451, Saudi Arabia; mtandevera@ksu.edu.sa

**Keywords:** pre-polymerized BisGMA, organic filler, dental composites, TEGDMA, degree of conversion, viscosity, thermal properties

## Abstract

Resin composite mimics tooth tissues both in structure and properties, and thus, they can withstand high biting force and the harsh environmental conditions of the mouth. Various inorganic nano- and micro-fillers are commonly used to enhance these composites’ properties. In this study, we adopted a novel approach by using pre-polymerized bisphenol A-glycidyl methacrylate (BisGMA) ground particles (XL-BisGMA) as fillers in a BisGMA/triethylene glycol dimethacrylate (TEGDMA) resin system in combination with SiO_2_ nanoparticles. The BisGMA/TEGDMA/SiO_2_ mixture was filled with various concentrations of XL-BisGMA (0, 2.5, 5, and 10 wt.%). The XL-BisGMA added composites were evaluated for viscosity, degree of conversion (DC), microhardness, and thermal properties. The results demonstrated that the addition of a lower concentration of XL-BisGMA particles (2.5 wt.%) significantly reduced (*p* ≤ 0.05) the complex viscosity from 374.6 (Pa·s) to 170.84. (Pa·s). Similarly, DC was also increased significantly (*p* ≤ 0.05) by the addition of 2.5 wt.% XL-BisGMA, with the pristine composite showing a DC of (62.19 ± 3.2%) increased to (69.10 ± 3.4%). Moreover, the decomposition temperature has been increased from 410 °C for the pristine composite (BT-SB0) to 450 °C for the composite with 10 wt.% of XL-BisGMA (BT-SB10). The microhardness has also been significantly reduced (*p* ≤ 0.05) from 47.44 HV for the pristine composite (BT-SB0) to 29.91 HV for the composite with 2.5 wt.% of XL-BisGMA (BT-SB2.5). These results suggest that a XL-BisGMA could be used to a certain percentage as a promising filler in combination with inorganic fillers to enhance the DC and flow properties of the corresponding resin-based dental composites.

## 1. Introduction

Resin composite mimics tooth tissues in structure and properties; thus, they are preferred nowadays in dentistry to restore/replace damaged or missing teeth. This composite quickly replaced traditional materials, such as amalgam, owing to their intriguing looks, improved clinical handling, and fewer adverse side effects [1,2]. Moreover, color customization to match the patient’s teeth is also critical in the adaptability and efficiency of resin composites [3]. The use of these composites is more gentle on the tooth structure because of their adhesive system, which allows composites to bond to the tooth structure without the need for extensive tooth preparation [4]. Resin composites comprise mainly four components: an organic polymer matrix (e.g., bisphenol A-glycidyl methacrylate (Bis-GMA), triethylene glycol dimethacrylate (TEGDMA), etc.), fillers, bonding agents, and an initiator. Nanocomposite reinforced with ceramic nano-fillers was expected to offer improved aesthetics, strength, and resilience [5,6,7]. Nevertheless, many studies indicate that most nanofillers offer only incremental improvements in mechanical properties [7,8], with some exceptions [9,10].

Bis-GMA and TEGDEMA blends are widely used for restorative dental materials [5,11,12]. Bis-GMA is an excellent base matrix for dental composites and offers significant advantages over dimethacrylate-based analogs, such as lower shrinkage, adequate mechanical strength, and good adhesion [13,14]. However, the key challenge of Bis-GMA is its high viscosity [13], which makes it challenging to incorporate a high filler content into the matrix resin. The viscosity of the matrix plays a significant role in improving specific properties of the dental composite, including filler content and degree of conversion (DC) [15,16]. Bis-GMA has the highest viscosity (910 Pa·s) among the commonly used di-methacrylate-based monomers owing to its high molecular mass and intermolecular interactions [17]. The molecular structure of Bis-GMA has a rigid central aromatic core and two hydroxyl groups that restrict kinetic motion and, consequently, reduce chain mobility. This makes it difficult to mix with other components and reduces the DC, therefore affecting the overall reliability and longevity of dental composites [18]. The DC is calculated from the ratio of polymerized double bonds converted to single bonds [19]. In resin composites, the DC is usually between 50 and 80%, which means that double bonds in the range of 20 to 50% are not converted [20]. A high DC causes high shrinkage during polymerization, while a low DC leads to poor mechanical properties, color stability, and biocompatibility [21]. Therefore, modifications of both organic and inorganic matrices are required to overcome these problems and achieve lower shrinkage and high DC.

Inorganic fillers with average particle sizes between 5 and 30 µm were once widely used but are now less so because of aesthetic and biocompatibility concerns. The fast abrasion of a soft resin matrix that protrudes or breaks away from the surface results in a rough surface, causing plaque formation and impairing the restoration’s aesthetics. The reinforcing filler’s particle size significantly impacts the dental composites’ surface roughness and polishability [22]. Nano-size fillers such as silsesquioxanes [23,24], organically modified silicates [25], and titania nanoparticles (NPs) have been added as fillers by researchers to enhance the performance of dental resin composites [26]. Generally, the small particles, due to their large specific surface area, dramatically enhance matrix-to-filler interaction and increase the resins’ viscosity while decreasing the achievable filler load [24,25,26]. Due to the hydrophilic surfaces and hydrogen bonding, silica NPs tend to adhere to each other and form irregular agglomerates. The agglomerates establish a network throughout the solution, affecting the resin’s rheological properties and causing a significant increase in viscosity while increasing silica loading. In order to incorporate high filler loading and reduce viscosity, researchers have used pre-polymerized resin and micro-fillers [27].

The current study aimed to evaluate the effect of pre-cured bisphenol A-glycidyl methacrylate ground particles (XL-BisGMA) as an organic filler in combination with inorganic silica NPs on the viscosity, degree of conversion, microhardness, and thermal properties. To the best of our knowledge, no prior research on using pre-polymerized BisGMA particles as filler in a dental composite is available. The null hypotheses were that adding various percentages of XL-BisGMA particles has no effect on the viscosity and DC. Moreover, no significant change in the microhardness and thermal properties has been observed by varying the concentration of XL-BisGMA.

## 2. Materials and Methods

### 2.1. Materials

TEGDMA (>95%), 2-(dimethylamino) ethyl methacrylate (DMAEMA; 98%), camphorquinone (CQ; 97%), BisGMA ( 98%), γ-MPS (98%), tetraethyl orthosilicate (TEOS, 98%), 2,2′-azobis(2-methylpropionitrile) (AIBN; ≥98.0%), and ethanol absolute (EtOH, ≥99.8%) were purchased from Sigma–Aldrich (Taufkirchen, Germany), and ammonium hydroxide (NH_4_OH, 35%) from Fisher Scientific (Loughborough, UK). Except AIBN, all the other chemicals were used in their original form. Distilled water was used wherever required.

### 2.2. Preparation of Prepolymerized BisGMA (XL-BisGMA)

**Figure 1:**
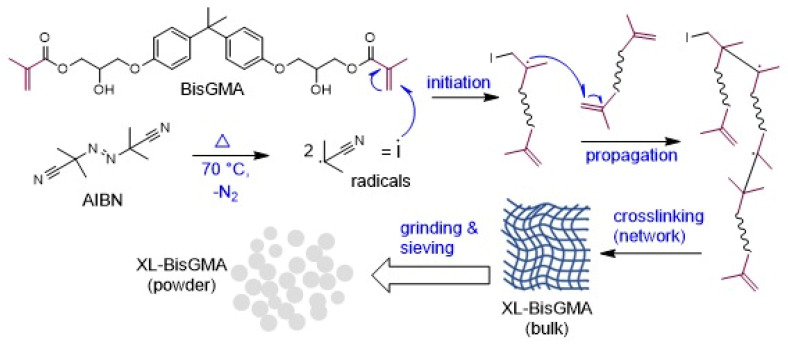
Synthesis of XL-BisGMA via thermal polymerization of BisGMA.

The pre-polymerized BisGMA particles were obtained using a bulk polymerization technique under an inert atmosphere and in the presence of AIBN as a thermal initiator. Figure 1 shows the synthesis of XL-BisGMA via thermal polymerization of BisGMA using AIBN as a thermal initiator. Briefly, BisGMA monomer was charged into a reflux setup of reaction flask, condenser, stirring and temperature-controlled hotplate, and nitrogen stream system. The thermal initiator AIBN was added as 0.1 wt.%, and after its complete dissolution in the monomer, the temperature for the reaction was increased to 70 °C. Typically, the free-radical polymerization mechanism occurs in three steps, i.e., initiation, propagation, and termination. The mixture was heated to 70 °C to allow the AIBN molecule to be thermally decomposed into two radicals. The generated radicals attack the double bond of the vinyl group to initiate the polymerization reaction (step (i)). Next, the monomeric radicals start to grow (propagation, step (ii)) by reacting with new BisGMA molecules. The BisGMA is a di-functional monomer, thus resulting in a highly crosslinked network. Finally, the reaction stops via one of the various termination processes (step (iii)) such as recombination, density effect, contamination, or monomer consumption. The bulky hard XL-BisGMA was then dried further, powdered, and sieved through a mesh strainer to obtain 11 µm particle sizes. The DC for XL-BisGMA was determined to be 95%. Section 2.5 explains the DC calculation.

### 2.3. Synthesis of Silanized Silica

Silica (0.5 µm in size) was surface functionalized with silane, as previously reported [28]. Thus, a solvent mixture consisting of water (40 mL), ethanol (250 mL), and ammonium hydroxide (25 mL) was prepared. The solution mixture was cooled in an ice bath, followed by dropwise addition of TEOS (45 mL) for 4–6 min. The reaction set was cooled to room temperature, followed by the addition of TEOS (33 mL) in EtOH (250 mL), and the reaction was carried out for 8 h. Then, the target coupling agent, γ-MPS (10 vol% concerning TEOS), was introduced and kept under stirring overnight. The resulting silica was separated by centrifugation followed by thorough washing using ethanol and drying under vacuum for 24 h.

### 2.4. Dental Composites Preparation

**Figure 2:**
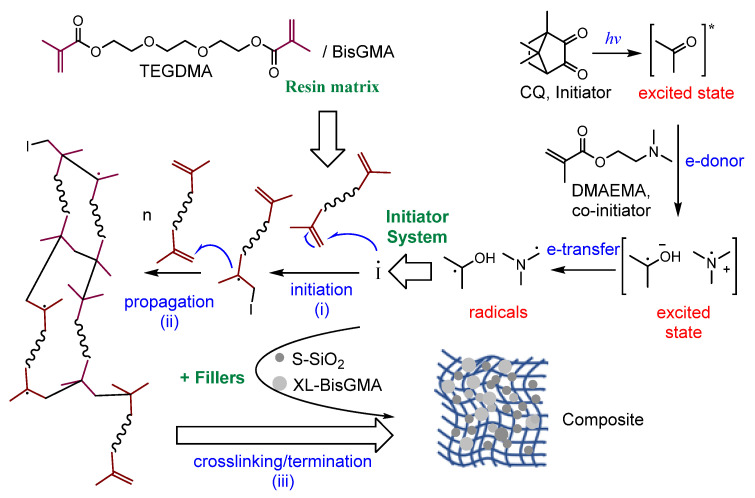
Reaction mechanism of the composite preparation, (*hν*) is the energy of light, (*) denotes excited state, (I) means initiator and dot (.) refers to radical, and (n) is a number of monomers unites.

Various dental composites comprised of 50% resin (1:1 ratio of BisGMA and TEGDMA) and 50% SiO_2_ filler with varying proportions of XL-BisGMA were prepared. The XL-BisGMA concentration was varied as 0.0, 2.5, 5, and 10% and designated as BT-SB0, BT-SB2.5, BT-SB5, and BT-SB10, respectively, as given in Table 1. The initiator was mixed with a blend of BisGMA/TEGDMA. The fillers were added and mixed manually, followed by mechanical mixing with an asymmetric double centrifuge at 3000 rpm, three times with a two-minute gap in between. The composite mixture was stored in dark containers at 8 °C. Specimens were prepared by carefully packing the composite resin into the mold and then placing a mylar strip with a cover plate on top of the composite. For complete curing of the specimen, the mold was irradiated for 60 s with a conventional light-curing unit (Bluephase, Ivoclar, Schaan, Lichtenstein) with 650 mW/cm^2^ intensity, 385 to 515 nm wavelength, and 10 mm adjustable wide light with homogeneous beam profile. The reaction mechanism for the conversion of C=C of the monomers into single C–C and preparation of the dental composite is illustrated in Figure 2. The reaction generally proceeds in the presence of a photo-initiator system, which commonly consists of an initiator (CQ) and a co-initiator (DMAEMA). Under light, the CQ transforms into an excited triplet state that is stabilized by the co-initiator amine group through electron/proton transfer to produce the desired radicals. The generated free radicals initiate a polymerization reaction in a similar way as described above for XL-BisGMA preparation. However, due to the presence of fillers (S-SiO_2_, XL-BisGMA) and additives, as well as the hardening phenomenon that restricts the radicals’ freedom, the reaction did not achieve a high DC. Yet, in dental applications, a DC higher than 55% is clinically acceptable [29].

### 2.5. Characterization

The viscosity of all the specimens was analyzed with a rheometer (MCR-72, Anton Paar, USA) in oscillation mode using a 25 mm parallel plate with a 0.5 mm gap and a frequency of 0.10–100 rad/s at 24 °C.

FTIR analysis of the functionalized silica and all composites was performed in the 4000–650 cm^−1^ range with a spectrometer (Nicolet-iS10, Thermo-Scientific, Waltham, MA, USA). The same setup was used to calculate the degree of conversion (DC) of all composites with an attenuated total reflection (ATR; diamond crystal). The DC was evaluated following a standard protocol for all test specimens [17]. Briefly, the specimen was placed between glass slides in a steel mold with a disc shape (5 × 2 mm) and light cured for one minute using an Elipar S10 curing device (3M ESPE-S10). The DC was obtained by calculating the specific peak areas of the spectrum representing the total mole ratio of the aliphatic (at 1637 cm^−1^) and aromatic (1608 cm^−1^) C=C before and after curing using Equation (1) [17].

(1) $$DC\;\left(\%\right)=\left[1-\frac{{\left(\frac{A_{1637}}{A_{1608}}\right)}_{cured}}{{\left(\frac{A_{1637}}{A_{1608}}\right)}_{uncured}}\right]\times 100$$

**Figure 3:**
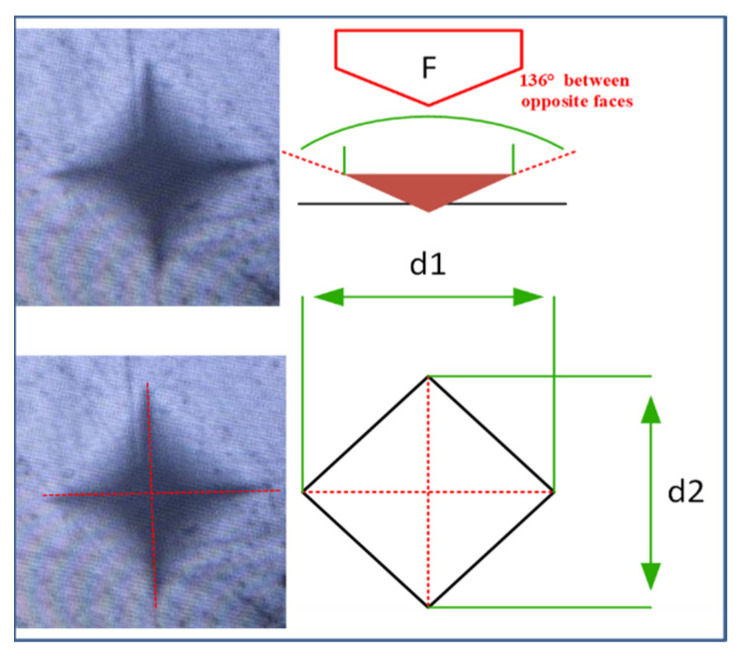
Indent area and diagonals during Vickers microhardness test.

A Vickers microhardness instrument (Innovatest) was used to measure each composite’s microhardness (MH) using 50 gf force for 15 s dwell time. The mean MH values of the pristine and pre-polymerized BisGMA composite were calculated using Equation (2). For all samples, three readings were taken. The indentation measurement was performed with the lens of the microscope having a magnification of 40×. The Vickers hardness was measured as per the following Equation (2):

(2) $$\mathrm{VH}=1.854\;\left(\frac{F}{D^{2}}\right)$$

where F represents the load (in kilograms-force) and D^2^ is the indent area (mm^2^), as depicted in Figure 3.

Thermogravimetric analysis (TGA) was carried out in air atmosphere at a ramp rate of 10 °C/min between 25 °C and 800 °C using a TA instrument Q50.

### 2.6. Statistical Analysis

The obtained results were statistically analyzed by SPSS (V 21.0, SPSS Inc., Chicago, IL, USA) using one-way analysis of variance (ANOVA) and Tukey post hoc tests. A *p* value ≤ 0.05 was considered as a significant difference among the groups (95% confidence level).

## 3. Results

### 3.1. Rheological Properties

**Figure 4:**
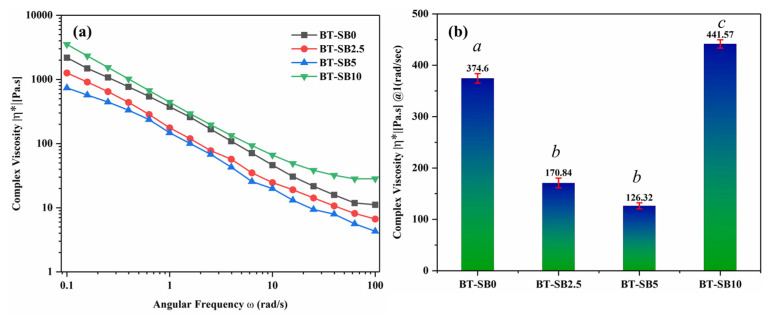
Complex viscosity of the composite groups in frequency range (0.1–100 rad/s frequency) at 25 °C: (**a**) complex viscosity vs. angular frequency; (**b**) complex viscosity of the various composites at 1 rad/s frequency (^a–c^ similar superscripts represent no significant difference).

The viscoelastic properties of the un-cured pristine composite (BT-SB0) and composites with varying concentrations of XL-BisGMA particles (BT-SB2.5, BT-SB5 and BT-SB10) are shown in Figure 4a. Figure 4b shows the complex viscosities of all test groups at a 1 rad/s frequency. It can be seen from the graph that the addition of a small amount of pre-polymerized BisGMA particles (2.5 wt.%) significantly reduced the complex viscosity from 374.6 (Pa·s) to 170.84 (Pa·s). Further increasing the XL-BisGMA content in the composite system (5 wt.%) followed the same trend and decreased the complex viscosity to 126.32. (Pa·s). However, increasing the XL-BisGMA content to 10 wt.% has a reverse impact on the complex viscosity. The addition of 10 wt.% XL-BisGMA (BT-SB10) increased the complex viscosity significantly from 126.32 Pa·s for BT-SB5 to 441.57 Pa·s.

### 3.2. FTIR Analysis and DC

**Figure 5:**
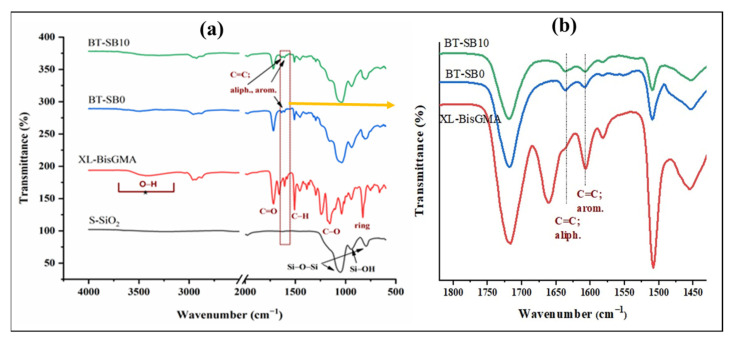
FTIR spectra (**a**) Silane functionalized SiO_2_ (S-SiO_2_), XL-BisGMA, BT-SB0, and BT-SB10 (**b**) Zoomed-in spectra of Xl-BisGMA, BT-SB0, and BT-SB10 showing the aliphatic and aromatic C=C peaks.

Figure 5a shows the FTIR spectra of the synthesized silanized silica (S-SiO_2_), XL-BisGMA, pristine composite group (BT-SB0), and XL-BisGMA composite (BT-SB10). As can be seen, the spectra of modified silica particles are accompanied by dominant peaks at 1051, 943, and 795 cm^−1^ assigned for Si-O-Si asymmetric stretching, Si-OH stretching, and Si-O-Si symmetric stretching vibrations, respectively [30]. The organic filler XL-BisGMA spectrum has shown the essential characteristic peaks for its monomer BisGMA [17]. However, the peak intensity of polymerization reaction canter at 1638 cm^−1^, the aliphatic C=C bonds, was diminished or almost disappeared, indicating complete conversion into single C–C bonds. Figure 5a also shows the spectrum of photo-cured BT-SB0 and BT-SB10 composites, those representing the XL-BisGMA free composite and XL-BisGMA-containing composite for comparison. The only difference that can be defined is the intensity of the aliphatic vinyl group, which is slightly lower for BT-SB10 than SB-BS0, supporting the slightly higher degree of conversion. Figure 5b depicts the zoomed-in spectra in the region 1450–1800 cm^−1^; no peaks for silica in this region are obtained. It can be observed that no peak for the aliphatic C=C in the XL-BisGMA spectra was obtained because of conversion to polymer, while the observed shoulder is possibly contributed to by C-H bending in the skeletal structure. By comparison of the composite spectra, the peak intensity of BT-SB10 at the aliphatic region around 1635 cm^−1^ is clearly higher than that for BT-SB0, confirming the calculated higher DC.

**Figure 6:**
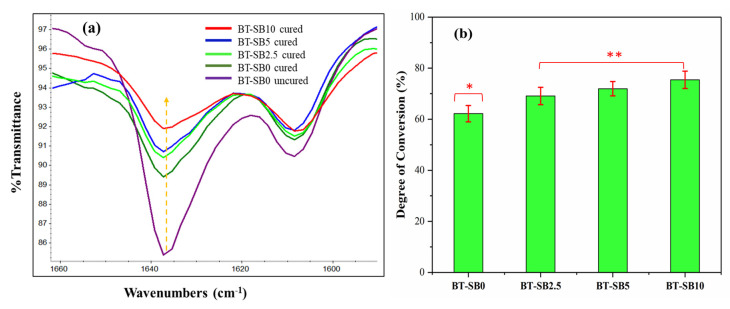
Illustration of the DC: (**a**) characteristic FTIR spectra with peak, (arrow direction represent the sequence), (**b**) change in DC% with respect to XL-BisGMA concentration; *, ** different symbols shows significant difference (*p* ≤ 0.05).

Figure 6a depicts the FTIR peak of the pristine (BT-SB0 uncured) and the composite groups with various concentration of XL-BisGMA (BT-SB2.5, BT-SB5, and BT-SB10) after curing (between 1660 and 1580 cm^−1^). From Figure 6a, it can be observed that the peak intensities of the vinyl group at 1637 cm^−1^ in the composites gradually decreased with increasing XL-BisGMA concentration, which represents the residual mole fraction of aliphatic C=C bonds [31,32], showing an enhanced DC with increasing concentration of XL-BisGMA in the composites. Figure 6b depicts the change in DC with respect to the amount of XL-BisGMA in the composites. The pristine composite group (BT-SB0) had the lowest DC (62.19 ± 3.2%). However, the addition of 2.5, 5, and 10 wt.% of the XL-BisGMA increased the DC to 69.10 ± 3.4, 71.94 ± 2.8, and 75.43 ± 3.4 respectively. Thus, the null hypothesis is rejected. The DC for a composite with a lower concentration of BT-SB2.5 was significantly higher (*p* ≤ 0.05) than the control group (BT-SB0). Further addition of XL-BisGMA resulted in a gradual increase in the DC; however, the difference was not significant (*p* > 0.05) among the groups. 

### 3.3. Microhardness

**Figure 7:**
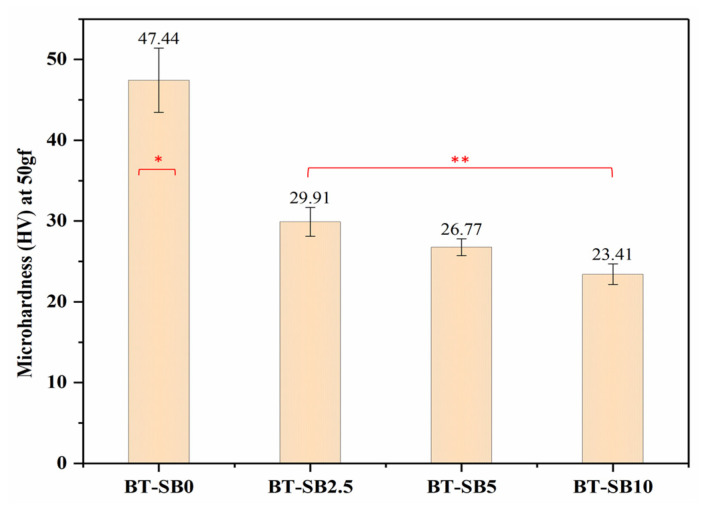
Vicker’s microhardness of the various XL-BisGMA resin composites. *, ** Different symbols shows significant difference (*p* ≤ 0.05).

Figure 7 shows the microhardness values of all the groups with and without pre-XL-BisGMA particles. Interestingly, it can be seen from Figure 7 that the incorporation of XL-BisGMA gradually decreased the microhardness. The pristine composite group (BT-SB0) resulted in the highest microhardness values (47.44 HV). However, the addition of 2.5 wt.% (BT-SB2.5) of XL-BisGMA significantly reduced (*p* ≤ 0.05) the microhardness value to 29.91 HV. Further addition of XL-BisGMA (BT-SB5, BT-SB10) did not induce statistically significant change in the microhardness (*p* > 0.05), as compared with BT-SB2.5. BT-SB10 showed the lowest microhardness value (23.41 HV) among the all the composite groups.

### 3.4. Thermal Gravimetric Analysis (TGA)

**Figure 8:**
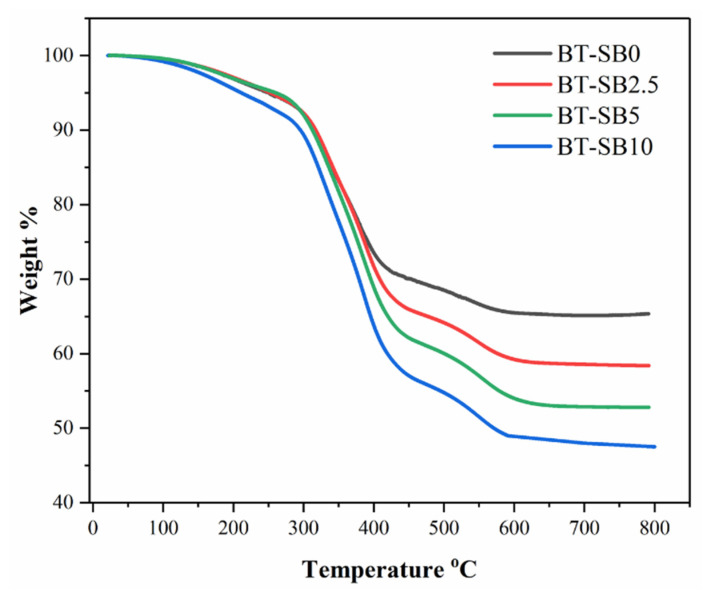
Thermal gravimetric analysis of the pristine and composites groups with XL-BisGMA particles.

Figure 8 shows the typical TGA thermograms of the pristine and XL-BisGMA composite groups. All the groups were tested from 25 to 800 °C at a heating rate of 10 °C min^−1^ in an air atmosphere. The TGA results revealed that for all composites groups, the TGA curves had demonstrated two-stage degradations between 350–450 °C and 450–600 °C. The decomposition temperature increased from 410 °C for BT-SB0 to 450 °C for BT-SB10 by increasing the XL-BisGMA content.

## 4. Discussion

Consideration of viscosity is important in the development of commercial dental materials because it determines the ease of handling of resin composites [33]. The effect of viscosity on reaction kinetics and DC in free-radical polymerization cannot be ignored because chain mobility is limited, and the rate of termination decreases at higher viscosities [34,35]. Many studies have investigated the effect of resin monomer composition on viscosity [20,36], but the effect of fillers on viscosity in dental composites is not so well described.

The current study evaluated the effect of organic filler (XL-BisGMA) on the viscosity of the dental composite using various concentrations. The viscosity graph in Figure 4a shows that by increasing the frequency, the complex viscosity was reduced in all test groups, indicating strong non-Newtonian behavior. The reduction in viscosity occurs because of the shear-thinning effect. The viscosity significantly decreases (*p* ≤ 0.05) with the addition of 2.5 wt.% XL-BisGMA (BT-SB2.5) and follows the reducing trend up to a 5 wt.% (BT-SB5) addition of XL-BisGMA. This response of resin mixture elucidates that the addition of XL-BisGMA changed the flowability of the resin/filler suspension, rejecting the null hypothesis. The reduction in complex viscosity could be attributed to the plasticizing behavior of XL-BisGMA, which increases polymer chain mobility and allows them to slide past each other. As a result, the viscosity of the mixture decreases. On the other hand, the high content of XL-BisGMA (10 wt.%) converted the suspension into a paste because of the saturation of the monomer chains, which prevented the chains from further absorbing XL-BisGMA filler and restricted the chains’ mobility [37]. 

Commercially, low-viscosity flowable-resin composites are considered as a special class of restorative materials. Low-viscosity resin composites have their own clinical significance. They can easily flow into the nooks and crannies in the cavity and adopt its shape. In addition, it has a better marginal adaptation, lower micro-leakage, and improved aesthetics because of easy polishability [38]. Therefore, in the current study, the reduction in viscosity caused by the addition of a XL-BisGMA will be clinically beneficial for marginal adaptation, flowability, and micro-leakage. 

The DC of the composite groups increased with increasing XL-BisGMA content (Figure 6b). The increase in DC at a low concentration of XL-BisGMA was significant (*p* ≤ 0.05) and is in line with the viscosity of the composite’s group (BT-SB2.5, BT-SB5), which corroborates that the increase in the DC is mainly due to the reduction in viscosity. However, at higher concentration (BT-SB10), the correlation of viscosity to DC is no longer valid and shows an opposite trend. Moreover, the change in the DC is not significant (*p* > 0.05). This may be due to the saturation and overlapping of the chains by achieving the maximum filler absorption capacity of the resin. The properties of dental resin composites are affected by their major constituents (resin composition, type, and concentration of fillers) and their interaction with each other. The nature and amount of each constituent in the dental composite significantly influence DC [39,40]. The current findings lead us to reject the null hypothesis because the DC of the resin composite was significantly increased (*p* ≤ 0.05) with a low percentage of XL-BisGMA, while, on the other hand, reducing the viscosity.

Microhardness is indicated by the resistance of a surface to penetration or permanent indentation. Microhardness is critical to the wear rate and clinical repair durability [41]. Surface hardness directly affects a material’s ductility, elasticity, toughness, and visco-elasticity. Moreover, abrasion and wear rate of dental composites determine the durability of clinical restorations [42]. Pre-polymerized fillers are used in micro-hybrid composites, and they wear and separate from the surface at a different rate than silica mineral fillers [43]. As a result, as the surrounding matrix degrades, the fillers are likely to be separated from the surface more easily, causing minimum changes in the matrix-to-filler ratio. The Vickers test is one of the most widely used in this regard to measure microhardness [44,45]. In general, the hardness of conventional composite materials is directly related to DC, which is elucidated by the higher density attained by the densely compacted network of dental composites [46,47,48,49]. However, it is not the only factor affecting the hardness of the dental composite. Other factors, including size, type, and nature of the fillers, have also been identified as more significant in affecting hardness. Micro-filled composite materials with particles of pre-polymerized organic filler and a high proportion of organic constituents are exceptions [22]. Pre-polymerized organic fillers have lower hardness than silica fillers and contain an organic component [41]. Therefore, in the current study, the reduction in the microhardness values could be mainly associated with an increase in the concentration of organic XL-BisGMA particles because of the organic nature of the filler [50]. The reduction in microhardness may be a clinical limitation for the XL-BisGMA resin composites because many properties of the dental composites are related to microhardness, including wear resistance, plaque accumulation, and durability [41]. However, with further modifications and combination with other fillers, the microhardness could be increased. On the other hand, some materials with high surface hardness may have lower flexural strength because of their brittle nature [51]. Therefore, microhardness may not be the only parameter to predict clinical failure. A balanced combination of surface hardness and other mechanical properties, on the other hand, will result in a clinically durable restoration. 

The TGA analysis demonstrated a two-stage degradation. Initially, a slight weight loss is observed between 150 and 250 °C, which may be attributed to the evaporation of the volatile groups, absorbed gases, moisture content, and homolytic scission of some chemical bonds in the polymeric composite network [52]. The primary decomposition and weight loss occur in the first stage between 350–450 °C, which may be attributed to the breakdown and decomposition of the organic backbone in the composite [53]. The second weight loss occurs between 450–600 °C and corresponds to the degradation of the aromatic benzene rings of the cured BisGMA with high C-C bond energy [54,55]. The values of the char yield decreased by increasing the pre-polymerized BisGMA particles, with BT-SB0 showing the highest char yield and BT-SB10 showing the lowest. This may be associated with reducing the concentration of silica NPs in the composites. On the other hand, the increase in decomposition temperature from 410 °C for BT-SB0 to 450 °C for BT-SB10 may be associated with the high degree of conversion and the abundance of the Benzene pendant group.

## 5. Conclusions

The addition of XL-BisGMA at a low concentration (2.5 wt.%) improved the flow properties of the BisGMA/TEGDMA/silica resin mixture, lowering the viscosity from 374.6 (Pa·s) to 170.84 (Pa·s). Furthermore, after light curing, the degree of conversion and thermal properties were increased while the surface microhardness was compromised. Within the scope of this study, we conclude that XL-BisGMA could be used as a potential inorganic filler for Bis-GMA/TEGDMA resin systems in combination with silica nanoparticles or other suitable fillers. However, more research and modifications are needed to investigate the true potential of XL-BisGMA as filler in dental composites.

## Figures and Tables

**Table 1:** Composition of the various dental composite groups.

Composite		Filler (wt.%)		Matrix (wt.%)		Initiator System (wt.%)	
No.	Code	Synthesized Silica	XL-BisGMA Powder	BisGMA	TEGDMA	Initiator (CQ)	Co-Initiator (DMAEMA)
1	BT-SB0	50	0	24.5	24.5	0.2	0.8
2	BT-SB2.5	47.5	2.5	24.5	24.5	0.2	0.8
3	BT-SB5	45	5	24.5	24.5	0.2	0.8
4	BT-SB10	40	10	24.5	24.5	0.2	0.8

## Data Availability

Not applicable.
